# Lung function and associations with multiple dimensions of dental health: a prospective observational cross-sectional study

**DOI:** 10.1186/s13104-016-2079-2

**Published:** 2016-05-17

**Authors:** Christian Henke, Stephan Budweiser, Rudolf A. Jörres

**Affiliations:** General Dental Practice, Mühldorf, Germany; Department of Internal Medicine III, Division of Pulmonary and Respiratory Medicine, RoMed Clinical Centre, Rosenheim, Germany; Institute and Outpatient Clinic for Occupational, Social and Environmental Medicine, Ludwig-Maximilians-University, Munich, Germany

**Keywords:** Periodontal disease, Oral health, Periodontitis, Lung disease, COPD

## Abstract

**Background:**

Epidemiological data suggest an association between respiratory diseases and periodontal health. However, the link between the overall dental status and single lung function measures, within a practical clinical context, is not well studied.

**Methods:**

Following a prospective cross-sectional design, consecutive adult patients were evaluated. Next to spirometry, anthropometric data, profession, smoking status, symptoms, self-rated exercise performance, comorbidities, allergies and medication were determined. Assessment of dental status comprised carious lesions, dental fillings, missing teeth, dentures, insufficient fillings/dentures, implants, oral mucosa diseases, calculus, decayed-missed-filling-teeth (DMF-T)-index, periodontal screening-index, and orthopantomograms.

**Results:**

Among 587 adult patients considered, 206 were included (119 female; median age 42.0 years; 56 % smoking history). Most patients had dental fillings (86.9 %), fix/mobile dentures (66.5 %), missing teeth (56.8 %) and calculus (84.0 %), the overall DMF-T being 15 (9; 21). Periodontitis was present in 53.9 %, an abnormal orthopanthomogram in 47.9 % of subjects. Regarding spirometric indices expressed as % predicted, dentures, missing teeth, oral mucosal diseases and a DMF-T > 15 (median) were associated with lower maximal expiratory flows at 25 % of vital capacity (MEF_25_) (p < 0.05 each). In adjusted logistic regression analyses, only dentures were associated with low MEF_25_ % predicted and with the ratio of forced expiratory volume in 1 s to forced vital capacity (FEV_1_/FVC; p < 0.05 each). However, periodontitis and DMF-T were linked to age (p < 0.001) and packyears (p < 0.05) only.

**Conclusion:**

Within a real-life clinical setting, only the presence of dentures showed weak associations with lung function, suggesting small airways dysfunction and obstruction. Most of the associations were explained by smoking habits and age.

## Background

Chronic obstructive pulmonary disease (COPD) is one of the most important chronic diseases worldwide [1], with a prevalence of about 5 % to more than 20 % in subjects aged ≥40 years [2, 3]. In addition to cigarette smoking, occupational exposures, indoor pollution, infections in early childhood, increasing age and low socioeconomic status have been identified as important risk factors for COPD [4].

There is evidence for an association between oral health and systemic and/or chronic diseases, particularly cardiovascular diseases, diabetes, osteoporosis, but also respiratory diseases such as COPD or pneumonia [5, 6]. Although difficult to prove, one hypothesis is that the oral cavity constitutes a potential source of pathogenetic micro-organisms that are transferred into the lower airways by micro-aspiration [7, 8]. Moreover, in patients with periodontitis, inflammatory mediators or proteases from saliva or a release of cytokines into the circulation, evoked by inflammatory oral processes, might affect the lower respiratory system [5], in analogy to cardiovascular disorders [9].

These considerations render a link between oral health and especially COPD plausible. Systemic inflammation and recurrent exacerbations of chronic inflammation, often due to bacterial infections, are of major importance for the progression of COPD. Conversely, the presence of COPD and its associated frequent infections could impair oral health [8], but such interactions could be substantially confounded by socioeconomic status, lifestyle, education, dental care, medication and smoking habits.

As yet, most data refer to the association between COPD and periodontal disease [10–17]. These studies have provided conflicting results and used different techniques, outcome measures or populations for the assessment of periodontitis. If there should be a relevant link between dental health and lung function, it would be reasonable to expect that oral bacteria and systemic inflammation lead to impaired respiratory function prior to the manifestation of COPD. Additionally it could be hypothesized that lung function is associated with other dimensions of dental health, e.g. caries and endodontic lesions [8].

Many of the investigations were retrospective [2–5, 7, 14, 18, 19] or differed with regard to study design and populations, methods and/or outcome measures used for dental assessment. Recently it has been reported that periodontal health (dental plaque, gingival bleeding and PD) was worse in smokers versus non-smokers, but in a multivariate model no major differences between smokers with versus without COPD occurred [18]. Similarly, in an earlier analysis of the NHANES-I population, no association was noted between any respiratory disease and the periodontal index [7]. In the larger NHANES III population comprising 7625 subjects, also no significant association between COPD and periodontitis has been found for former and non-smokers, except for current smokers [19].

Based on this it was concluded that the risk to develop periodontitis is predominately explained by smoking. In contrast, clinical attachment loss (CAL), PD, and number of missing teeth were found to be associated with reduced lung volumes, airflow limitation and hyperinflation but not with diffusing capacity [10]. Moreover, in very severe COPD, chronic marginal periodontitis was more frequent than in other severe respiratory diseases [14]. In line with this, CAL, PD and an oral hygiene test were higher in COPD patients as compared to a control population, after adjustment for covariates [11]. In a Chinese population plaque index was the most important periodontal factor related to COPD [12]. As a result, the most recent meta-analysis stated a significant association between periodontitis and COPD but also a substantial heterogeneity between studies [20]. Moreover, the funnel plot was markedly asymmetrical, which is considered to be indicative of a publication bias.

As a further relevant issue, the majority of studies focused on the presence of COPD as defined by a fixed FEV_1_/FVC <70 % [11, 12, 19, 21–23] or did not report lung function in detail [7, 14, 24]. In the present study, we covered all common spirometric lung function measures and did not restrict the analysis to the mere presence of COPD. We also explored the associations with dental health by using spirometric measures in absolute or % predicted values, applying established and, if possible, the novel GLI reference values. In particular we included MEF_50_ and MEF_25_ which are traditionally considered as being related to small airway dysfunction and indicative for the onset of obstructive lung disease. This was complemented by a comprehensive assessment of dental health as far as maximally feasible under the conditions of routine clinical practice.

We investigated the possible link between lung function and several dimensions of dental health beyond the periodontal status in a prospective “real-life” setting. For this, spirometry as well as a comprehensive, standardized dental examination by established techniques in dental practice were performed. We also included the decayed-missed-filled-teeth (DMFT)-index as an established score of dental health [25] which never has been explored regarding respiratory diseases in adults.

## Methods

### Study Protocol

Following the study protocol, consecutive, potentially eligible subjects consulting a general dental practice in Mühldorf, Bavaria, Germany, from July 2011 to January 2013 were asked for study participation. Inclusion criteria were age ≥18 years and written informed consent. Subjects with known chronic infections (e.g. HIV or tuberculosis) or malignant diseases were excluded. We recorded anthropometric data and assessed medical history by routine dental exploration and a standardized questionnaire. Patients were asked for pulmonary symptoms (cough, sputum production, wheezing), comorbidities (pulmonary, cardiovascular, other), medication, the presence of type I allergies, smoking status (current, previous, packyears), the profession including possible workplace exposures, self-estimated exercise capacity, dyspnoea level, and previous periodontal treatment.

Spirometry (Masterscope/Labmanager; Carefusion, Höchberg, Germany) was performed according to the guidelines of the American Thoracic Society (ATS) [26] and evaluated using established [27] and, when possible, the novel global lungs initiative (GLI) reference values [28]. Forced expiratory volume in 1 s (FEV_1_), forced vital capacity (FVC), peak expiratory flow (PEF) and maximal expiratory flow rates at 50 and 25 % (MEF_50_ and MEF_25_) of vital capacity were recorded. Clinically relevant bronchial obstruction was assumed at a ratio of FEV_1_/FVC <70 % or, alternatively, <lower limit of normal (LLN) according to GLI. The spirometry was done by one single trained dental practice technician.

### Dental health assessment

The comprehensive dental examination was also done by the same experienced dentist who performed the spirometry and who assessed the following items with regard to the presence and the number of respective teeth: carious lesions, dental fillings, missing teeth; dentures (fix, mobile, implants), insufficient fillings/dentures; presence of oral mucosal diseases (fistula, aphthous ulceration, gingivitis) and calculus. Finally, the DMFT-index was determined (with the highest value of 28, as third molars are not considered) [25].

Periodontal status was evaluated by the periodontal screening-index (PSI) using an established WHO-probe (DB767R, PCP-11.5C, Aesculap, B. Braun, Melsungen, Germany) with a marker of length and a hemisphere at its end. The probe was introduced cautiously in four positions (mesiobuccal, distobuccal distopalatinal, mesiopalatinal) of each single tooth and probing depth (PD), bleeding and roughness of surface evaluated. Codes were chosen as follows [29]: (0) black marker (PD 3.5 mm) completely apparent during probing, no calculus and/or insufficient edge of restauration occur, gums are healthy, no bleeding after probing; (1) black marker completely apparent during probing, no calculus and/or insufficient edge of restauration occur, minor bleeding after probing; (2) black marker completely apparent during probing, manifest calculus and/or insufficient edge of restauration occur; (3) black marker only partially apparent during probing, any other previously mentioned criteria may exist; (4) black marker disappearing completely during probing (PD > 5.5 mm).

For documentation the maxilla and mandible was divided in sextants: maxillary (S1-S3, right-posterior, front, left-posterior) and mandibular (S4–S6, left-posterior, front, right-posterior), and the highest value was registered for each sextant. Periodontal status was categorized as follows: 0 in all sextants: no periodontitis; 1 or 2: no significant periodontitis but gingivitis; 3: moderate periodontitis; 4: severe periodontitis. The presence of periodontitis was defined according the following criteria: PSI ≥ 3 or previous treatment of periodontitis, or bone loss ≥1.

X-rays (orthopanthomogram), if available or required for medical reasons (e.g. dental surgery, search for apical lesions/dental focal points), were evaluated with regard to apical lesions, exposed furcation, displaced teeth, and horizontal loss of alveolar bone. The horizontal loss of alveolar bone was categorized as follows: grade 1: bone loss <1/3 of alveolar bone; grade 2: bone loss >1/3 but <2/3, or grade 3: bone loss >2/3 of alveolar bone. Following the study protocol, approved by the ethics committee of the Ludwig-Maximilians-University, Munich, Germany (study number 066/11), no X-rays were taken only for the study. Only if there was a medical reason for the comprehensive dental examination, new X-rays were taken.

### Statistical analyses

Data were continuously documented and finally analyzed with SPSS software (IBM, Version 19.0, Chicago, IL, USA). Due to skewed distributions (Shapiro–Wilk Test) continuous variables were summarized as median values (quartiles), while using the non-parametric Mann–Whitney U test to assess intergroup differences. In case of categorical variables the Fishers exact test was applied (two-sided). Bivariate correlation analyses (Pearson or Spearman) were employed to assess associations between spirometric measures and patients’ characteristics. For the identification of independent associations with dental health, a logistic (dichotomous variables) or multivariate linear regression analysis was performed, whereby the single dimensions of dental health were used as dependent variables. Smoking, parameters being significant regarding the respective dimension of dental health, and single lung function indices (one by one) were introduced as explanatory variables. When using % predicted values of lung function we did not additionally introduce age, gender and height, as these were already taken into account by the calculation of % predicted values. P values <0.05 were considered statistically significant.

## Results

### Study population

Among the 578 adult patients asked for study participation, 206 were included; 372 patients refused participation, delivered insufficient spirometry, or were excluded according to the study protocol. Anthropometric data, comorbidities, profession, job-related exposures, smoking history, respiratory symptoms, and exercise capability are shown in Table 1. Regarding medication, most often prescribed were thyroxine/iodine (n = 19; 9.2 %), angiotensin-converting enzyme inhibitors and β-blockers (each n = 7; 3.4 %).

**Table 1:** Patients’ characteristics of the total population (n = 206)

Parameter	
Age (years)	42.0 (32.0; 54.7)
BMI (kg/m^2^)	24.6 (21.8; 27.6)
Gender (f)	119 (57.8 %)
Current smoking	59 (28.6 %)
Previous smoking	58 (28.2 %)
History of smoking	117 (56.8 %)
Pack years	10.0 (4.0; 20.7)
Cough	28 (13.6 %)
Sputum production	11 (5.3 %)
Wheezing	4 (1.9 %)
Reduced performance	45 (21.8 %)
NYHA class (≥I)	11 (5.3 %)
Asthma (self-reported)	21 (10.2 %)
Systemic hypertension	20 (9.7 %)
Cardiovascular disease	9 (4.4 %)
Diabetes	5 (2.4 %)
Hypothyreosis	19 (9.2 %)
Osteoporosis	4 (1.9 %)
Allergy type I	42 (20.2 %)
Academic profession	23 (11.2 %)
Job-related exposure	27 (13.1 %)

Results are shown either as n (%) or median (quartiles)

*BMI* body-mass-index; *NYHA* New York Heart Association

### Lung function

Overall, subjects showed a FEV_1_ of 96.0 (87.6; 105.5) % predicted (GLI), a FVC of 100.0 (91.1; 108.9) % predicted (GLI) and a ratio of FEV_1_/FVC 78.9 (73.9; 83.2) % (Table 2).

**Table 2:** Spirometric measures of the total population

Parameter	n	Median (quartiles)
FEV_1_ (L)	206	3.20 (2.75; 3.82)
FEV_1_ (% predicted)	206	102.6 (92.2; 112.4)
FEV_1_ (% predicted)^GLI^	206	96.0 (87.6; 105.5)
FVC (L)	206	4.06 (3.51; 4.98)
FVC (% predicted)	206	109.9 (100.8; 119.2)
FVC (% predicted)^GLI^	206	100.0 (91.1; 108.9)
FEV_1_/FVC (%)	206	78.9 (73.9; 83.2)
FEV_1_/FVC (% predicted)^GLI^	206	96.7 (91.1; 101.8)
PEF (L/s)	206	6.98 (5.95; 8.65)
PEF (% predicted)	206	95.0 (84.7; 107.1)
MEF_50_ (L/s)	150	3.22 (2.51; 4.18)
MEF_50_ (% predicted)	150	71.3 (58.7; 92.0)
MEF_25_ (L/s)	150	0.94 (0.52; 1.34)
MEF_25_ (% predicted)	150	50.0 (31.6; 68.2)

GLI reference values according to Global Lungs Initiative [18]

*FEV*_*1*_ forced expiratory volume in 1 s; *FVC* forced vital capacity; *PEF* peak expiratory flow; *MEF*_*50*_ maximal expiratory flow at 50 % of vital capacity. *MEF*_*25*_ maximal expiratory flow at 25 % of vital capacity

Among the 206 patients, 27 (13.1 %) had FEV_1_/FVC < 70 % and 25 (12.1 %) < LLN. When applying the GOLD criteria, 17 (GLI 13) subjects (8.3 %, GLI 6.3 %) had COPD I, and 10 (GLI 14) (4.9 %, GLI 6.8 %) COPD II.

There were inverse correlations between the number of packyears and FEV_1_ (L) (r = −0.189; p = 0.007), FEV_1_ (% predicted, GLI) (−0.176; p = 0.011), FEV_1_/FVC (%) (r = −0.309; p < 0.001), FEV_1_/FVC (% predicted, GLI) (−0.221; p = 0.001), MEF_50_ (L) (r = −0.241; p = 0.003), MEF_50_ (% predicted) (r = −0.227; p = 0.005), MEF_25_ (L) (r = −0.338; p < 0.001) and MEF_25_ (% predicted) (r = −0.336; p < 0.001). Age was inversely correlated to absolute values of FEV_1_ (r = −0.440), FVC (r = −0.329), FEV_1_/FVC (r = −0.307), MEF_50_ (r = −0.363), MEF_25_ (r = −0.570; p < 0.001 each), PEF (−0.152; p = 0.030). Regarding % predicted values only MEF_50_ (r = −0.167; p = 0.041) and MEF_25_ (r = −0.304; p < 0.001) were significant.

### Dental health

Most patients had dental fillings (86.9 %), fix/mobile dentures (66.5 %), missing teeth (56.8 %) or calculus (84.0 %), and 97.5 % a DMFT-index > 0; overall (n = 206) it was 15 (9; 21) (Table 3). Regarding PSI, 24 subjects (11.7 %) had no periodontitis (index 0), 32 (15.5 %) index 1, 59 (28.6 %) index 2 (gingivitis only), 51 (24.8 %) index 3 (moderate periodontitis), and 39 (18.9 %) index 4 (severe periodontitis); one patient did not have any teeth. Thus, 90 (43.7 %) showed a PSI-index of ≥3, median 2.0 (1.0; 3.0). Moreover, 59 (28.6 %) had previous periodontal treatment. Thus, periodontitis was present in 111 patients (53.9 %). Among those with orthopanthomograms (n = 167), 34 (20.4 %) showed exposed furcations, 34 (20.4 %) apical lesions, and 69 (41.3 %) alveolar bone loss (53 patients grade I, 16 grade II), 39 participants had no X-ray or evaluable X-ray documents. Thus, radiologic abnormalities of any kind were observed in 80 subjects (47.9 %).

**Table 3:** Basic dental status of the total population (n = 206)

Variable	n	%	Number of teeth^a^
Carious lesions	82	39.8	1.5 (1.0; 3.0)
Dental fillings	179	86.9	7.0 (4.0; 11.0)
Dentures (fix or mobile)	137	66.5 %	–
Dentures fix	133	64.6 %	7.0 (3.0; 12.0)
Dentures mobile	26	12.6 %	–
Implants	22	10.7	1.0 (1.0; 3.5)
Insufficient fillings/dentures	57	27.7	2.0 (1.0; 2.5)
Missing teeth	117	56.8	3.0 (2.0; 8.5)
Disease of oral mucosa (any)	36	17.5	–
Apthen	3	1.5	–
Fistula	11	5.3	–
Gingivitis	30	14.6	–
Calculus	173	84.0	–
DMF-T (>0)	201	97.6	15 (9.5; 21)

*DMF*-*T* Decayed-Missed-Filled-Teeth-index

^a^Referring to subjects affected, presented as median (quartiles)

### Association between periodontitis and spirometry

Subjects with *periodontitis* (n = 111) had higher age, BMI, weight and more packyears (Table 4), but less type-I allergies. Moreover, the absolute values of FEV_1_, FVC, FEV_1_/FVC and MEF_25_ were significantly lower (Table 5), while this was not the case for % predicted values.

**Table 4:** Patients’ characteristics in relation to the presence of periodontitis

Parameter	No periodontitis		Periodontitis		p value
Age (years)	95	32.7 (25.2; 40.9)	111	51.8 (41.5; 61.6)	<*0.001*
Height (m)	95	1.70 (164; 1.76)	111	1.70 (1.65; 1.79)	0.379
Weight (kg)	95	67 (60; 82)	111	76 (63; 89)	*0.006*
BMI (kg/m^2^)	95	23.7 (21.0; 26.3)	111	25.4 (22.5; 29.0)	*0.006*
Gender (f)	95	61 (64.2 %)	111	59 (53.2 %)	0.163
Current smoking	95	28 (29.5 %)	111	31 (27.9 %)	0.877
Previous smoking	95	21 (22.1 %)	111	37 (33.3 %)	0.088
History of smoking	95	49 (51.6 %)	111	68 (61.3 %)	0.204
Pack years	95	0.3 (0.0; 5.5)	111	5.0 (0.0; 18.8)	*0.002*
Cough	95	9 (9.5 %)	111	19 (17.1 %)	0.153
Sputum production	95	3 (3.2 %)	111	8 (7.2 %)	0.230
Wheezing	95	0 (0.0 %)	111	4 (3.6 %)	0.126
Reduced performance	95	15 (15.8 %)	111	30 (27.0)	0.063
NYHA class (≥I)	95	3 (3.2 %)	111	8 (7.2)	0.230
Asthma (self-reported)	95	10 (10.5 %)	111	11 (9.9 %)	0.532
COPD (FEV_1_/FVC < 0.7)	95	9 (9.5 %)	111	19 (17.1 %)	0.153
Systemic hypertension	95	5 (5.3 %)	111	15 (13.5 %)	0.059
Cardiovascular disease	95	2 (2.1 %)	111	7 (6.3 %)	0.182
Diabetes	95	1 (1.1 %)	111	4 (3.6 %)	0.377
Hypothyreosis	95	7 (7.4 %)	111	12 (10.8 %)	0.473
Osteoporosis	95	1 (1.1 %)	111	3 (2.7 %)	0.626
Allergy type I	95	42 (44.2 %)	111	29 (26.1 %)	*0.008*
Academic profession	95	13 (13.7 %)	111	10 (9.0 %)	0.375
Job-related exposure	95	13 (13.7 %)	111	14 (12.6 %)	0.490

Defined by PSI ≥ 3 or previous treatment of periodontitis or alveolar bone loss ≥ 1

Italic characters are used for statistically significant comparisons (p < 0.05)

*BMI* body-mass-index; *NYHA* New York Heart Association; *COPD* chronic obstructive pulmonary disease; *FEV*_*1*_ forced expiratory volume in 1 s; *FVC* forced vital capacity

**Table 5:** Spirometric data in relation to the presence of periodontitis

Parameter	No periodontitis		Periodontitis		p value
FEV_1_ (L)	95	3.42 (2.97; 3.88)	111	3.05 (2.53; 3.75)	*0.001*
FEV_1_ (% predicted)	95	100.9 (91.9; 112.7)	111	102.8 (92.3; 112.3)	0.580
FEV_1_ (% predicted)^GLI^	95	95.9 (88.9; 107.1)	111	96.7 (86.5; 105.3)	0.847
FVC (L)	95	4.24 (3.70; 5.04)	111	3.88 (3.41; 4.98)	*0.025*
FVC (% predicted)	95	108.6 (99.6; 118.6)	111	110.9 (101.9; 121.0)	0.333
FVC (% predicted)^GLI^	95	101.1 (91.2; 110.4)	111	99.6 (90.3; 107.3)	0.384
FEV_1_/FVC (%)	95	79.9 (76.0; 84.2)	111	77.8 (72.4; 82.2)	*0.008*
FEV_1_/FVC (% predicted)^GLI^	95	95.7 (91.3; 100.0)	111	97.6 (90.9; 102.9)	0.263
PEF (L/s)	95	7.01 (6.18; 8.73)	111	6.80 (5.71; 8.61)	0.252
PEF (% predicted)	95	94.3 (83.5; 106.0)	111	95.0 (85.7; 109.2)	0.469
MEF_50_ (L/s)	70	3.28 (2.73; 4.22)	80	3.12 (2.37; 4.16)	0.240
MEF_50_ (% predicted)	70	70.9 (61.5; 92.7)	80	74.3 (53.6; 91.3)	0.928
MEF_25_ (L/s)	70	1.07 (0.80; 1.50)	80	0.84 (0.44; 1.24)	*0.003*
MEF_25_ (% predicted)	70	52.6 (38.4; 73.0)	80	46.5 (28.8; 63.2)	0.067

Defined by PSI ≥ 3 or previous treatment of periodontitis or alveolar bone loss ≥ 1

Italic characters are used for statistically significant comparisons (p < 0.05)

See Table 1

In multivariate logistic regression analyses taking the presence of periodontitis as dependent variable together with the absolute value of FEV_1_, FVC, FEV_1_/FVC or PEF (one by one), only age (p < 0.001) and packyears (p < 0.05) were statistically significant. When introducing the absolute value of MEF_50_/MEF_25_, only age remained significant (p < 0.001). In multivariate analyses including % predicted values, only packyears (p ≤ 0.01 each) were significantly associated with periodontitis.

### Association between basic dental status including DMF-T and spirometry

Regarding specific characteristics (Table 3), subjects with *dental fillings* (n = 179) showed higher absolute values of FEV_1_ (p = 0.020), FVC (p = 0.007), PEF (p = 0.002), height and better exercise performance (p < 0.05 each). Lung function indices as % predicted were not significantly different.

Patients with *dentures* had lower absolute values of FEV_1_ (p < 0.001), FVC (p = 0.002), FEV_1_/FVC, MEF_50_, MEF_25_, and MEF_25_ % predicted (p < 0.001 each), but higher age (p < 0.001) and were more often current smokers (p = 0.034).

Subjects with *missing teeth* (n = 117) had lower absolute values for FEV_1_ (p < 0.001), FVC (p = 0.001), FEV_1_/FVC (p = 0.017), MEF_50_ (p = 0.003), MEF_25_ (p < 0.001) and MEF_25_ % predicted (p = 0.021), but higher age (p < 0.001), BMI (p = 0.024) and more packyears (p = 0.008). Moreover they more often showed hypertension, hypothyreosis, cough, reduced exercise performance (p < 0.05 each), and previous smokers history (p = 0.009).

Subjects with *oral mucosaldiseases* (n = 36) had lower values of MEF_25_ % predicted (p = 0.025), more often male gender (p = 0.005) and reduced exercise performance (p = 0.028). Patients with *calculus* showed lower FEV_1_/FVC (p = 0.026), higher age (p = 0.006), BMI (p = 0.008), weight (p = 0.021) and more often hypertension (p = 0.049). Lung function did not significantly differ between patients with versus without carious lesions, insufficient fillings/dentures and implants.

In adjusted bivariate logistic regression analyses dentures were significantly associated with MEF_25_ % predicted (Table 6), and marginally failed statistical significance when also including age (p = 0.067). No statistical associations with lung function indices (absolute or % predicted) were found in adjusted multivariate analyses including dental fillings, missing teeth, oral mucosal disease, calculus, carious lesions, insufficient fillings/dentures and implants.

**Table 6:** Association of dentures and MEF_25_ % predicted using bivariate regression analysis

Variable	Coefficient B	P value	Exp (B) (95 % CI)
MEF_25_ % predicted	−0.029	*<0.001*	0.972 (0.958–0.986)
Current smoking	−1.165	*0.004*	0.312 (0.140–0.693)

Italic characters are used for statistically significant comparisons (p < 0.05)

*MEF*_*25*_ maximal expiratory flow at 25 % of vital capacity, *Exp (B)* odds ratio

Subjects with a DMF-T index ≥15 (median; n = 105) versus those with lower values were characterized by a higher age (p < 0.001), more packyears (p = 0.001), more often systemic hypertension, previous smoking history, and impaired exercise performance (p < 0.05 each). Regarding spirometry, the absolute values of FEV_1_, FVC, FEV_1_/FVC, MEF_50_, MEF_25_, and MEF_25_ % predicted were significantly lower in patients with a DMF-T-index≥ 15 (Table 7).

**Table 7:** Spirometric data in relation to the DMF-T index

Parameter	DMF-T < 15		DMFT ≥ 15		p value
FEV_1_ (L)	101	3.43 (3.03; 3.86)	105	2.97 (2.43; 3.76)	<*0.001*
FEV_1_ (% predicted)	101	100.9 (92.9; 112.4)	105	103.0 (91.0; 112.7)	0.829
FEV_1_ (% predicted)^GLI^	101	95.4 (88.7; 105.4)	105	97.1 (86.4; 105.5)	0.912
FVC (L)	101	4.24 (3.81; 5.18)	105	3.78 (3.32; 4.83)	<*0.001*
FVC (% predicted)	101	109.0 (99.6; 118.7)	105	110.6 (102.2; 121.3)	0.512
FVC (% predicted)^GLI^	101	101.3 (91.1; 109.8)	105	99.6 (90.4; 108.2)	0.493
FEV_1_/FVC (%)	101	79.8 (75.3; 84.2)	105	77.8 (73.0; 82.0)	*0.017*
FEV_1_/FVC (% predicted)^GLI^	101	95.6 (91.3; 100.1)	105	97.8 (90.9; 102.5)	0.263
PEF (L/s)	101	7.03 (6.20; 8.89)	105	6.74 (5.73; 8.06)	0.113
PEF (% predicted)	101	93.9 (83.9; 105.4)	105	95.1 (85.3; 110.4)	0.246
MEF_50_ (L/s)	75	3.38 (2.83; 4.29)	75	2.82 (2.11; 4.06)	*0.012*
MEF_50_ (% predicted)	75	71.6 (62.4; 92.6)	75	69.9 (51.2; 91.3)	0.382
MEF_25_ (L/s)	75	1.10 (0.81; 1.50)	75	0.73 (0.43; 1.15)	<*0.001*
MEF_25_ (% predicted)	75	55.1 (39.8; 73.6)	75	42.5 (29.1; 62.2)	*0.014*

Comparing subjects with DMF-T index either < versus ≥ 15 (median)

Italic characters are used for statistically significant comparisons (p < 0.05)

*DMF*-*T* decayed-missed-filled-teeth-index, for other abbreviations see Table 1

In a bivariate logistic regression analysis, taking DMF-T index (either < versus ≥ 15) as dependent variable together with the absolute of FEV_1_, FVC, FEV_1_/FVC or PEF, again only age (p < 0.001) and packyears (p < 0.05) were significant. When introducing absolute values of MEF_50_/MEF_25_, only age remained significant (p < 0.001). In multivariate analyses including % predicted values, packyears were still significant (p ≤ 0.01 each). In a multivariate regression analysis, taking DMF-T as dependent variable, similar results were obtained.

### Association between abnormal radiologic findings and spirometry

Subject with (n = 80) versus without radiologic abnormalites those were more often males (p = 0.011), had higher BMI (p = 0.024), weight (p = 0.006), age (p < 0.001) and more packyears (p = 0.010). Regarding spirometry, the absolute values of FEV_1_, FEV_1_/FVC and MEF_25_ were significantly (p < 0.01 each) lower in patients with abnormal radiologic findings.

In an adjusted bivariate logistic regression analysis, taking the presence of any radiologic finding as dependent variable, significant association were only detected for age (p < 0.001 each) when including absolute values, or packyears (p < 0.05 each) when including % predicted values.

### Association between abnormal dental health and the presence of COPD

Patients with versus without FEV_1_/FVC <70 % or <LLN did not differ with regard to periodontitis (Table 4), the DMF-T index or the other single dimensions of dental health. However, these patients more often were male, had more packyears and current smoking history (p < 0.05 each). In adjusted multivariate analyses, FEV_1_/FVC < 70 % was significantly associated with the presence of dentures (Table 8).

**Table 8:** Association of dentures and FEV_1_/FVC < 70 % using bivariate regression analysis

Variable	Coefficient B	p value	Exp (B) (95 % CI)
FEV_1_/FVC < 70 %	1.208	*0.026*	3.346 (1.156–9.687)
Current smoking	−0.862	*0.011*	0.422 (0.217–0.821)
gender (f/m)	0.153	0.627	1.166 (0.628–2.164)

Italic characters are used for statistically significant comparisons (p < 0.05)

*FEV*_*1*_ forced expiratory volume in 1 s; *FVC* forced vital capacity; *Exp (B)* odds ratio

The major results have been presented at the annual meeting of the European Respiratory Society 2014 [30].

## Discussion

The present study is the first prospective cross-sectional study evaluating the associations between spirometric measures and multiple dimensions of dental health in a random population of adult patients from a general dentist’s practice. Using % predicted values, the associations of the different indices of dental health with lung function appeared to be rather weak. While patients with dentures, missing teeth, oral mucosal diseases and a higher DMF-T-index presented with lower MEF_25_, in adjusted multivariate analyses, only the presence of dentures was significantly associated with MEF_25_ % predicted and FEV_1_/FVC < 70 %. Particularly periodontitis and DMF-T were mainly linked to age and smoking.

As expected we found strong correlations between age and dental health, particularly regarding periodontitis and the composite DMF-T index. There was also a consistent association with smoking habits, in line with the literature [18, 19]. In contrast, no significant associations between periodontitis and spirometric measures were present after adjustment for covariates. Recently, Holfreter and colleagues [10] found that measures of periodontitis were significantly linked to nearly all dynamic and static lung function indices. However, these were retrospective analyses, and dental health and lung function were assessed at different time points. The associations were clearly weaker when smoking was included as covariate. Moreover, the adjustment for body height reduced the correlations of periodontitis with lung function considerably. Therefore authors hypothesized that smaller persons might have a higher susceptibility to develop severe periodontitis. However, their findings could also be interpreted in the way that the use of % predicted values of lung function, correcting for age, gender and height for each individual index, probably would have resulted in less strong associations.

In the present analysis we also explored the associations between other dimensions of dental health beyond periodontitis. Most spirometric indices expressed in absolute values were significantly different with regard to specific characteristics of dental health (carious lesions, dentures, missing teeth, oral mucosa disease, calculus). Using % predicted values, dentures, missing teeth, oral mucosa diseases and a DMF-T-index > median were associated with lower MEF_25_ only. In further adjusted multivariate analyses, accounting for the difference between groups, only dentures were significantly linked to MEF_25_ % predicted. Considering a potential causal relationship between reduced lung function and impaired dental health, it seems pathophysiologically plausible that the association is particularly reflected in the small airways where the initial changes in obstructive respiratory diseases occur [31]. Interestingly, Bergström and colleagues [18] recently found a weak but significant correlation between the occurrence of emphysema in CT scan or reduced carbon monoxide diffusing capacity and pocket depth and/or teeth loss. They hypothesized that this points towards an association between dental health and lung pathology not reflected in spirometry. Unfortunately they did not assess MEF_50_ or MEF_25_, which are capable of detecting minor spirometric airway abnormalities.

The association between dentures and lung function has been described in the latest literature by Linuma and co-workers [6]. Some authors described associations between missing or remaining teeth and lung function [6, 10] or the presence of COPD [12, 18, 22, 23]; these associations were not significant in our analysis after adjustment for covariates. From a dentist’s perspective, both findings are not unexpected as dentures or missing teeth often reflect poor overall dental health status. As known, carious lesions acquired in early childhood or dentistry phobia often leads to early tooth loss and dentures; this phenomena is particularly observed in socially disadvantaged families [32–34]. This also fits to studies which found a link between oral hygiene status and COPD [11, 24, 35]. Surprisingly, the introduction of the composite DMF-T index did not enhance the associations in the present analysis, although patients with a higher DMF-T-index showed worse MEF_25_ % predicted values. The associations probably have been weakened by the “F” (=fillings) subscore, which reflects a restored condition and not so much an inflammatory focus potentially linked to lung function impairment. In line with this, patients with dental fillings did not have worse lung function.

Oral mucosal disease was also associated with MEF_25_ % predicted, but this association again was not significant after adjustment. This was most likely to the fact that the overall number of patients having oral mucosal diseases was rather low. In a larger population, gingival index was associated with airway obstruction in former smokers [21]. Moreover, a significant inverse correlation between gingival index and FEV_1_ % predicted was recently reported in patients with COPD after adjustment for covariates [11].

Only few authors have considered radiologic abnormalities assessed by orthopantomogramms with regard to lung function; these studies addressed alveolar bone loss with regard to the presence of COPD. In the investigation by Wang and co-workers [22], the higher alveolar bone loss in COPD marginally failed statistical significance in univariate analysis, whereas in another study [12] it was very well associated with COPD. Similarly, Leukfeld and colleagues [14] observed that a mean marginal bone level ≥4 mm was associated with COPD independent of other risk factors for periodontitis. We found radiologic abnormalities in 47.9 % of subjects but no significant associations with lung function. Similar results were obtained when including patients with alveolar bone loss of ≥1 only (41.3 %). Possibly, radiologic abnormalities only occur in very advanced respiratory disease [14], but not in a general population as studied by us.

### Limitations

An obvious limitation is the relatively low number of study participants. Many patients were not interested in lung function testing, medical research, or could not perform spirometry adequately. However, we consider it likely that this happened randomly and that we still investigated a representative sample. Since the study was performed in a dentist’s practise, we had to dispense from postbronchodilator measurements. Thus, the presence of COPD via FEV_1_/FVC < 70 % might have been overestimated. MEF_50_ and MEF_25_ could not be determined in all patients, however a full determination would presumably enhance the significance of the associations. As the study is observational, it does not allow inferring causal relationships. The determination of serum inflammatory markers or oral bacterial colonization would have been desirable regarding potential interactions between lung and dental health, although in a recent analysis fibrinogen and high-sensitive C-reactive were not suggestive of links between systemic inflammation and lung function [10]. We characterised patients comprehensively with regard to dental health, but the protocol had to remain feasible and we might have missed some factors. Actually, tooth brushing times and method, experience of dental floss use, dental visits and oral health knowledge have been demonstrated to be significantly related to the risk of COPD [14, 22]. We could not adjust the analysis for these factors, similarly as, e.g., Holfreter and collegues in their impressive analysis [10]. Whether the inclusion of the profession (academic versus non-academic) or school education [10] is sufficient to account for these factors, is unclear.

## Conclusion

In conclusion, this prospective cross-sectional study assessed the associations between dental health and spirometric measures in a general real-life population, using a broad panel of dental health measures. The major result was that dentures, missing teeth, oral mucosa diseases and a higher DMFT were weakly linked to impaired age-adjusted indices of small airways dysfunction in terms of MEF_25_ % predicted, while dentures were the only dimension remaining significant in multivariate analyses. Our data suggest that at least phenomenologically, the link between lung function and dental health is weak, compared to the needs for relevant predictions in individuals. Further prospective studies could benefit from the information on specific assessments of dental health provided by our study. These studies might also address respiratory disorders beyond the diagnosis of COPD, particularly with regard to small airway dysfunction.

## Abbreviations

BMI: body-mass-index
NYHA: New York Heart Association
FEV_1_: forced expiratory volume in 1 s
FVC: forced vital capacity
PEF: peak expiratory flow
MEF_50_: maximal expiratory flow at 50 % of vital capacity
MEF_25_: maximal expiratory flow at 25 % of vital capacity
GLI: reference values according to global lungs initiative
DMF-T: decayed-missed-filled-teeth-index
COPD: chronic obstructive pulmonary disease
PSI: periodontal screening-index
ATS: American Thoracic Society
PEF: peak expiratory flow
LLN: lower limit of normal
WHO: World Health Organization
PD: probing depth
S1-S3: sextants maxillary (S1-S3, right-posterior, front, left-posterior)
S4-S6: sextants mandibular (S4-S6, left-posterior, front, right-posterior)
GOLD: global Initiative for chronic obstructive Lung disease
NHANES: National Health and Nutrition Examination Survey
CAL: clinical attachment loss
CT: computed tomography
CPITN: community periodontal index of treatment needs

## References

[1] Mannino DM, Buist AS (2007). Global burden of COPD: risk factors, prevalence, and future trends. Lancet.

[2] Buist AS, McBurnie MA, Vollmer WM, Gillespie S, Burney P, Mannino DM, Menezes AMB, Sullivan SD, Lee TA, Weiss KB, Jensen RL, Marks GB, Gulsvik A, Nizankowska-Mogilnicka E (2007). International variation in the prevalence of COPD (the BOLD Study): a population-based prevalence study. Lancet.

[3] Menezes AMB, Perez-Padilla R, Jardim JRB, Muiño A, Lopez MV, Valdivia G, de Montes Oca M, Talamo C, Hallal PC, Victora CG (2005). Chronic obstructive pulmonary disease in five Latin American cities (the PLATINO study): a prevalence study. Lancet.

[4] Salvi SS, Barnes PJ (2009). Chronic obstructive pulmonary disease in non-smokers. Lancet.

[5] Cullinan MP, Ford PJ, Seymour GJ (2009). Periodontal disease and systemic health: current status. Aust Dent J.

[6] Linuma T, Arai Y, Abe Y, Takayama M, Fukumoto M, Fukui Y, Iwase T, Takebayashi T, Hirose N, Gionhaku N, Komiyama K (2015). Denture wearing during sleep doubles the risk of pneumonia in the very elderly. J Dent Res.

[7] Scannapieco FA, Papandonatos GD, Dunford RG (1998). Associations between oral conditions and respiratory disease in a national sample survey population. Ann Periodontol.

[8] Didilescu AC, Skaug N, Marica C (2005). Respiratory pathogens in dental plaque of hospitalized patients with chronic lung diseases. Clin Oral Investig.

[9] Tonetti MS, D’Aiuto F, Nibali L, Donald A, Storry C, Parkar M, Suvan J, Hingorani AD, Vallance P, Deanfield J (2007). Treatment of periodontitis and endothelial function. N Engl J Med.

[10] Holtfreter B, Richter S, Kocher T, Dörr M, Völzke H, Ittermann T, Obst A, Schäper C, John U, Meisel P, Grotevendt A, Felix SB, Ewert R, Gläser S. Periodontitis is related to lung volumes and airflow limitation - a cross-sectional study. Eur Respir J. 2012. **(Epub ahead of print)**. 10.1183/09031936.00109112 23222882

[11] Peter KP, Mute BR, Doiphode SS, Bardapurkar SJ, Borkar MS, Raje DV. Association between periodontal disease and chronic obstructive pulmonary disease-a reality or just a dogma. J Periodontol. 2013. [Epub ahead of print]. 10.1902/jop.2013.120347 23339345

[12] Si Y, Fan H, Song Y, Zhou X, Zhang J, Wang Z (2012). Association between periodontitis and chronic obstructive pulmonary disease in a Chinese population. J Periodontol.

[13] Kucukcoskun M, Baser U, Oztekin G, Kiyan E, Yalcin F (2013). Initial periodontal treatment for prevention of chronic obstructive pulmonary disease exacerbations. J Periodontol.

[14] Leuckfeld I, Obregon-Whittle MV, Lund MB, Geiran O, Bjørtuft Ø, Olsen I (2008). Severe chronic obstructive pulmonary disease: association with marginal bone loss in periodontitis. Respir Med.

[15] Deo V, Bhongade ML, Ansari S, Chavan RS (2009). Periodontitis as a potential risk factor for chronic obstructive pulmonary disease: a retrospective study. Indian J Dent Res.

[16] Scannapieco FA, Ho AW (2001). Potential associations between chronic respiratory disease and periodontal disease: analysis of National Health and Nutrition Examination Survey III. J Periodontol.

[17] Offenbacher S, Beck JD, Barros SP, Suruki RY, Loewy ZG (2012). Obstructive airway disease and edentulism in the atherosclerosis risk in communities (ARIC) study. BMJ Open..

[18] Bergström J, Cederlund K, Dahlén B, Lantz A, Skedinger M, Palmberg L, Sundblad B, Larsson K (2013). Dental health in smokers with and without COPD. PLoS One.

[19] Hyman JJ, Reid BC (2004). Cigarette smoking, periodontal disease: and chronic obstructive pulmonary disease. J Periodontol.

[20] Zeng X, Tu M, Liu D, Zheng D, Zhang J, Leng W (2012). Periodontal disease and risk of chronic obstructive pulmonary disease: a meta-analysis of observational studies. PLoS One.

[21] Katancik JA, Kritchevsky S, Weyant RJ, Corby P, Bretz W, Crapo RO, Jensen R, Waterer G, Rubin SM, Newman AB (2005). Periodontitis and airway obstruction. J Periodontol.

[22] Wang Z, Zhou X, Zhang J, Zhang L, Song Y, Hu FB, Wang C (2009). Periodontal health, oral health behaviours, and chronic obstructive pulmonary disease. J Clin Periodontol.

[23] Zhou X, Han J, Song Y, Zhang J, Wang Z (2012). Serum levels of 25-hydroxyvitamin D, oral health and chronic obstructive pulmonary disease. J Clin Periodontol.

[24] Sharma N, Shamsuddin H (2011). Association between respiratory disease in hospitalized patients and periodontal disease: a cross-sectional study. J Periodontol.

[25] Klein H, Palmer CE, Knutson JW (1938). Studies on dental caries. Dental status and dental needs of elementary school children. Public Health Rep.

[26] American Thoracic Society 1995 (1994). Standardization of Spirometry. Update. Am J Respir Crit Care Med.

[27] Quanjer PH, Tammeling GJ, Cotes JE, Pedersen OF, Peslin R, Yernault JC (1993). Lung volumes and forced ventilatory flows. Report working party standardization of lung function tests, european community for steel and coal. Official statement of the European Respiratory Society. Eur Respir J Suppl.

[28] Quanjer PH, Hall GL, Stanojevic S, Cole TJ, Stocks J (2012). Age- and height-based prediction bias in spirometry reference equations. Eur Respir J.

[29] ADA and AAP introduce dentists to new time saving periodontal evaluation system. Va Dent J. 1992. 69(Suppl 4):16–17. 1308335

[30] Henke C, Jörres R, Budweiser S (2014). Lung function and associations with multiple dimensions of dental health. ERJ.

[31] McDonough JE, Yuan R, Suzuki M, Seyednejad N, Elliott WM, Sanchez PG, Wright AC, Gefter WB, Litzky L, Coxson HO, Paré PD, Sin DD, Pierce RA, Woods JC, McWilliams AM, Mayo JR, Lam SC, Cooper JD, Hogg JC (2011). Small-airway obstruction and emphysema in chronic obstructive pulmonary disease. N Engl J Med.

[32] Holst D, Schuller AA (2012). Oral health in a life-course: birth-cohorts from 1929 to 2006 in Norway. Community Dent Health.

[33] Riley JL, Gilbert GH (2005). Childhood dental history and adult dental attitudes and beliefs. Int Dent J.

[34] Pearce MS, Steele JG, Mason J, Walls AWG, Parker L (2004). Do circumstances in early life contribute to tooth retention in middle age. J Dent Res.

[35] Liu Z, Zhang W, Zhang J, Zhou X, Zhang L, Song Y, Wang Z (2012). Oral hygiene, periodontal health and chronic obstructive pulmonary disease exacerbations. J Clin Periodontol.

